# Simulation and test of a V-shaped seed-guiding surface and concave pit furrow opener for seed displacement restriction: Verification on seeding uniformity and post-soil-contact seed motion limitation

**DOI:** 10.1371/journal.pone.0337695

**Published:** 2025-12-19

**Authors:** Guoqiang Dun, Yang He, Xinxin Ji, Chunyu Ma, Xinming Li, Luhan Wang

**Affiliations:** 1 Intelligent Agricultural Machinery Equipment Engineering Laboratory, Harbin Cambridge University, Harbin, China; 2 College of Mechanical and Electrical Engineering, Northeast Forestry University, Harbin, China; China Construction Fourth Engineering Division Corp. Ltd, CHINA

## Abstract

In plot breeding tests, sowing uniformity directly impacts the accuracy of crop genetic improvement trials. Existing furrow openers often cause spacing fluctuations and lateral seed drift due to structural limitations. To address this, a sharp-angle roller furrow opener with independently rotatable V-shaped ball rollers was innovatively designed based on traditional sharp-angle flat-bottom openers, meeting soybean sowing agronomic requirements. The V-shaped ball rollers form V-grooves and spherical pit rows at the furrow bottom through rolling, with ridge-like structures between pits effectively limiting seed longitudinal displacement and rolling jumps upon soil contact, thus improving sowing uniformity. Performance verification was conducted via discrete element simulation and soil trench testing: An EDEM-based soybean seed-soil-opener interaction model was built, using spacing fluctuation coefficients, lateral offset dispersion, and average lateral offset as indexes to compare the new opener with sharp-angle sharp-bottom and flat-bottom openers. Three opener prototypes were 3D-printed and tested on a self-made soil trench bench. Both simulation and bench test results showed that under identical conditions, the new opener significantly reduced spacing fluctuations and lateral offsets, outperforming traditional designs in sowing uniformity and enhancing breeding trial reliability. This study constructs a sustainable agricultural furrow opener design framework through structural innovation and dual experimental validation, integrating mechanical performance optimization with resource-environmental benefits, and provides a new technological pathway for clean production concepts in agricultural equipment.

## 1. Introduction

Soybean is an important oil crop in the world. Therefore, it is of great practical significance to increase soybean production. Planting better soybean varieties can increase soybean yields without increasing the acreage planted. Plot breeding tests provided a direct and intuitive understanding of the quality status of different strains of seed from various soil environments, as well as the most effective method for selecting superior seed [1]. In plot breeding tests, sowing is the most fundamental and crucial part of the test, and the quality of sowing is a significant factor influencing the test’s accuracy [2].

Numerous studies on crops, such as soybeans, have demonstrated that metrics like the standard deviation of seed spacing, which reflects the uniformity of seed distribution, have a direct impact on crop yield. Uniform seeding densities maximize the space available for each plant to grow and reduce inter-plant competition, which affects yield [3–5]. Therefore, the use of plot breeding seeders instead of manual precision seeding in plot breeding tests can enhance the precision and efficiency of seeding, thereby increasing the scientificity and reliability of the test [6].

In addition to the seeding performance of the Seed metering device of the plot seeding planter, the quality of seed-soil contact optimization technology will also affect seeding accuracy. The unreasonable shape of the seed furrow increases the degree of rolling and bouncing of the seed after it comes into contact with the soil, resulting in an uneven distribution of the seed within the seed furrow [7,8]. Therefore, it is essential to enhance the uniformity of seed distribution in the seed furrow by researching and designing a furrow-opening device, which is crucial for improving the performance of plot breeding planters.

Many scholars’ research on furrow openers has focused on optimizing the relevant parameters of the original furrow openers to achieve the effect of reducing forward resistance, reducing soil adhesion, and reducing soil disturbance. Aikins et al. [9] selected two types of straight furrow openers and four types of bent-leg furrow openers and conducted a comparative study by varying the operating speed and depth. The EDEM simulation revealed that the bent-leg furrow openers exhibited a slight increase in furrow width under various operating speed conditions, thereby providing better control over soil movement and enhancing the efficiency and quality of seeding. James et al. [10] Designed a curved leg furrow opener, which was validated using EDEM simulation to demonstrate that the curved leg furrow opener reduces soil disturbance and exhibits better adaptability to varying operating depths compared to the straight leg furrow opener. Song et al. [11] used a tine furrow opener as a research object and measured the area and rate of soil disturbance by this type of furrow opener using equipment such as a soil-trough experimental bench, and simulated the experiment using discrete element simulation, which showed that the range of soil disturbance decreases with the increase of the distance from the furrow opener.

Some scholars have designed new types of furrow openers by applying bionic methods and also obtained excellent operational performance. Jia et al. [12] designed and processed a sliding furrow opener based on the canine curve of the dog badger. Experiments demonstrated that the optimized sliding furrow opener significantly reduced operational resistance compared to standard core-share furrow openers. The coefficients of variation for furrow depth, furrow width, side-row height, and side-row width of the furrows opened were all less than 5%. Wang et al. [13] designed a bionic coupled disk furrow opener based on the convex shells of the pangolin’s fecal thorax and the posterior ridge scales. Under the same experimental conditions, the furrow opening resistance of this furrow opener was significantly smaller than that of the ordinary flat disk furrow opener. It was substantially smaller than that of ordinary flat disk furrow openers. Sun et al. [14] Optimized the design of bionic disc furrow openers based on bionic principles, achieving a significantly better drag reduction rate compared to conventional disc furrow openers. The drag reduction rate reached 15.36% at a furrow opening speed of 0.6 m/s and a soil moisture content of 20%.

To address issues such as poor furrow formation quality and inconsistent sowing depth, scholars have conducted in-depth research into structural innovations and performance optimization of the coulter. Zhao et al. [15] addressed the issue of loose seedbeds at the bottom caused by traditional double-disc seed drills by designing a serrated seed drill. Field trials demonstrated that this design creates a seedbed with a firm base and loose top layer, reducing the coefficient of variation in seed spacing by 60.81%. Jia et al. [16,17] addressed the shortcomings of conventional seed drills through a series of innovative designs: to reduce lateral soil displacement and improve seed distribution uniformity, they proposed a contour-following sliding-blade seed drill, reducing the coefficient of variation for lateral seed spacing and sowing depth by 32.52% and 10.88% respectively; To optimize seed furrow formation quality, a double-V-shaped coulter was designed. This effectively minimized soil disturbance, produced uniformly shaped seed furrow cross-sections, and significantly enhanced sowing precision and seedbed establishment. Hou et al. [18] focused on a double-disc coulter as their research subject. By optimizing its key structural parameters, they achieved a more uniform soil flow field distribution during operation. The coefficient of variation for sowing depth decreased by 43.5%, while that for lateral seed spacing variation reduced by 66.6%, significantly enhancing the operational quality of precision sowing. Although structural innovations or parameter optimization have effectively enhanced the seedbed preparation capability of furrow openers under ridge cultivation conditions, dedicated furrow-opening devices tailored to precision seeding requirements still necessitate further systematic research.

However, there is limited research on furrowing devices for constructing a seed furrow structure suitable for precision sowing, which, to some extent, restricts the widespread application and development of precision sowing technology. Therefore, this study presents a sharp-angle spherical roller furrow opener for precision seeding of soybeans that utilizes a roller with a spherical protrusion to roll and squeeze a seed furrow, characterized by a V-shaped furrow and evenly spaced spherical pits at the bottom of the seed furrow. This special seed furrow structure reduces the bouncing and rolling displacement of soybean seeds after they come into contact with the soil, resulting in uniform sowing and improved reliability of the plot breeding test.

The performance of the sharp-angled roller spherical roller furrow opener was validated through comparative testing of discrete element simulation and comparative testing of the soil bin, which provides references for improving the seeding quality of plot breeding planters.

## 2. Materials and methods

### 2.1. General structure of the furrow opener

To address the problem of the existing furrow opener not effectively limiting seed displacement after seeds fall into the soil in plot breeding tests (which leads to uneven sowing), a sharp-angle spherical roller furrow opener—consisting of a left side plate, a right side plate, a furrowing blade, a V-shaped spherical roller, and a bearing—was designed in this study. The furrow opener is designed with a soil-return guiding notch at the lower rear of each side plate. The front lower end is equipped with a furrowing blade securely fixed by threaded fasteners, and a V-shaped spherical roller is mounted via bearings between the side plates at the rear portion of the furrowing blade.

**Fig 1:**
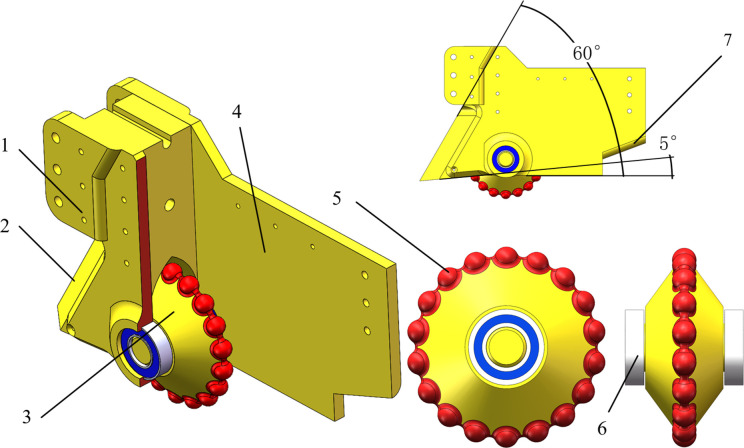
Sharp-angle spherical roller furrow opener overall structure picture. 1. Left side plate 2. Furrowing blade 3. V-shaped spherical roller 4. Right side plate 5. Ball-shaped bulge 6. Bearing 7. Soil-return guiding notch.

During seeding operations, the furrow opener, a component that directly contacts the soil, has structural parameters critically influencing the seed furrow structure. Among these parameters, the rake angle and penetration clearance angle are the primary determinants of its performance. Studies have shown that when the penetration clearance angle is too large, the furrow opener is subjected to greater forward resistance, which in turn affects the quality of the backfill. Conversely, a too small an angle reduces the performance of soil penetration. The forward resistance of the furrow opener increases with the increase of the rake angle, which is linear, while the vertical resistance has the opposite trend, but the vertical resistance is smaller compared to the forward resistance; a smaller rake angle reduces the strength of the furrow opener and the depth of the furrow opening [19–21]. The furrow opener’s penetration angle was set at 60°, and the penetration clearance angle was set at 5°. The furrow opener can press a spherical pit in the seed furrow using the V-shaped spherical roller with 18 spherical projections evenly distributed circumferentially. Measurements of multiple soybean varieties by previous researchers indicate that their sphericity generally exceeds 85%, with three-dimensional dimensions concentrated between 6–8 mm. Their shape approximates a sphere with a diameter of 6–8 mm [22,23]. To ensure soybean seeds can settle into spherical depressions within seed furrows, the spherical protrusions on the roller are designed as spheres with a diameter of 9 mm. The overall structure of the sharp-angle spherical roller furrow opener is shown in Fig 1.

### 2.2. Working process of furrow opener

During operation, the furrow blade first displaces soil laterally, creating a flat-bottomed seed trench with consistent width maintained by parallel side panels. The V-shaped spherical roller then contacts the bottom of the seed furrow, which the furrow opener has opened. It extrudes a uniformly arranged spherical pit at the bottom of the seed furrow by a ball-shaped bulge. The portion of the bottom of the seed furrow that is not completely compressed forms a ridge-like structure, which reduces the longitudinal displacement of the seed. The seed guide tube, positioned behind the V-shaped spherical roller, releases seeds into the trench. Seeds either drop directly into the pits or are guided into them via the inclined walls of the V-shaped pits, ensuring precise placement of the seeds. After seeding is completed, the soil’s self-weight is utilized to bring the soil along the soil-return guiding notches, which are designed at the rear of the side panels to achieve the first layer of soil coverage over the seed.

**Fig 2:**
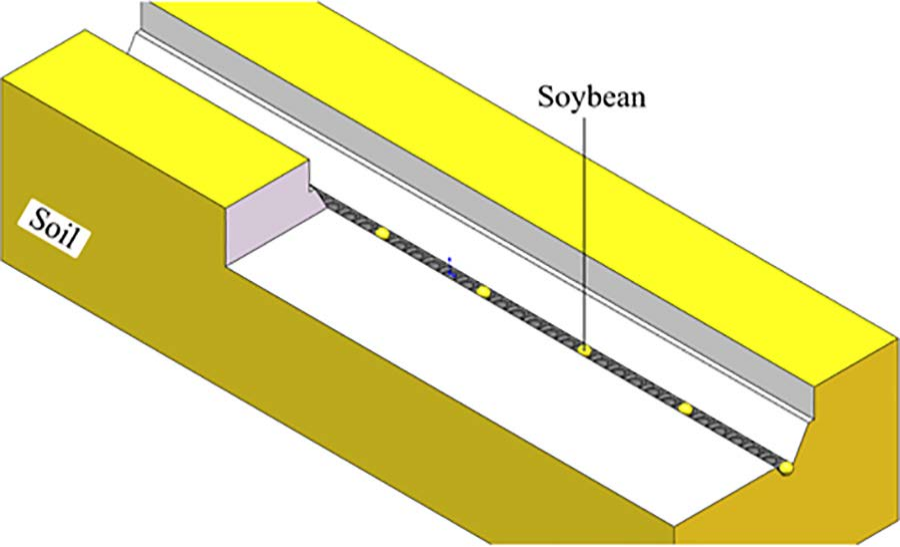
Theoretical furrow profile created by the furrow opener.

Since the spherical pit is formed by extrusion, the surrounding soil becomes more compact, creating a soil environment conducive to seed germination. The seed furrow structure created by the furrow opener guides the seed to fall into the pit and effectively limits lateral and longitudinal displacement of the seed after it falls into the soil using a ridge-like structure and a spherical pit. The theoretical furrow profile created by the furrow opener is shown in Fig 2.

### 2.3. Comparative testing of discrete element simulation

To ensure the feasibility of the simulation, the following assumptions are made for the simulation test:

(1) Simplify the actual soil into a sufficient number of particles and set the relevant parameters of the soil and soybean seeds according to the relevant literature.
(2) Soybean seeds are generated from a seed guide tube at regular intervals and are given a certain initial velocity of descent.
(3) The working process of the furrow opener involves imparting a certain speed to the furrow opener, allowing it to act on the soil particles.
(4) The furrow opener and the simulation container (soil bin) are considered rigid bodies, with their elastic deformation and wear being ignored in actual work.
(5) The simulation conditions for the comparison tests were identical, differing only in the structure of the furrow opener model.
(6) Selection of the Hertz-Mindlin contact model for particle contact.
(7) The simulation does not account for the effect of different soil types on soil cohesion resulting from changes in moisture dynamics.

#### 2.3.1. Particle models.

According to related research, the common soybean seed (He Nong 60) was selected as the reference model for soybeans [24]. The soybean simulation model was set as a spherical particle with a radius of 3.5 mm, a density of 1272.15 kg/m³, a Poisson’s Ratio of 0.23, and a shear modulus of 63 MPa [25,26].

Since the number of soil particles in the simulation experiment is large, the setting of the soil particle size has a significant influence on the result and the simulation test time. If the particle size is too small, the number of particles will increase, resulting in a substantial increase in simulation test time. Conversely, if the particle size is too large, the realism of the simulation test will be compromised. According to the literature, setting the radius of soil particles to 2–3 mm ensures the accuracy of the simulation and meets the computer’s performance requirements [26]. In this study, the soil particles were modeled as spherical with a radius of 2 mm, a density of 2600 kg/m³, a Poisson’s Ratio of 0.3, and a shear modulus of 50 MPa [27,28].

#### 2.3.2. Furrow opener models.

**Fig 3:**
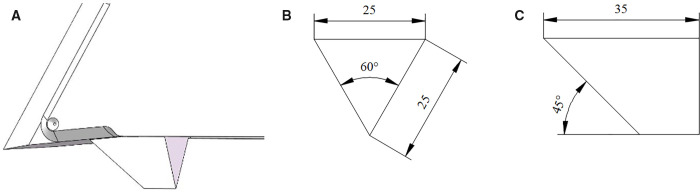
The dimensions of the sharp-angle protrusion. (a) Schematic diagram of the location of the sharp-angle protrusions. (b) Main view. (c) Right view (all dimensions are in mm).

To verify the improvement in the structure’s effect on seeding accuracy, the sharp-angle flat-bottom furrow opener (a prototype of the sharp-angle spherical roller furrow opener) and the sharp-angle pointed-bottom furrow opener (which features a sharp-angle protrusion at the bottom of the sharp-angle flat-bottom furrow opener) were selected as comparison models. The dimensions of the sharp-angle protrusion are shown in Fig 3. The three models were identical except for their bottom structures.

**Fig 4:**
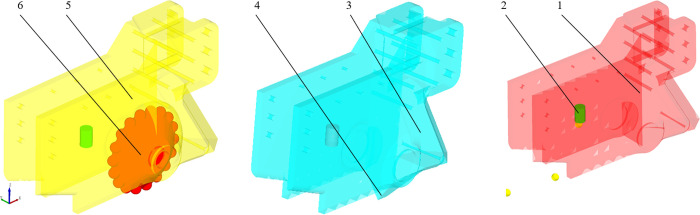
Three-dimensional modeling of furrow openers. 1. Sharp-angle flat-bottom furrow opener 2. Seed guide tube 3. Sharp-angle pointed-bottom furrow opener 4. Sharp-angle protrusion 5. Sharp-angle spherical roller furrow opener 6. V-shaped spherical roller.

According to the simplification principle of numerical simulation, the actual furrow opener is simplified by removing components that are not related to the operating process. A simplified 3D model of the 3 types of furrow openers was built using SolidWorks at a scale of 1:1 and imported into the Geometry item of EDEM in IGES format. The simulation model of the 3 types of furrow openers is shown in Fig 4.

To achieve the synergistic verification of digital simulation and physical experiments, this study employs the principle of parameter consistency design. The furrow opener material is set as PLA with a density of 1,290 kg/m³, a Poisson’s Ratio of 0.3, and a shear modulus of 10.4 MPa [29]. The finalized simulation parameters are shown in Table 1.

**Table 1:** Simulation parameters.

Property	Value
Density of soil (kg/m^3^)	2600
Density of soybean (kg/m^3^)	1272.15
Density of PLA (kg/m^3^)	1290
Poisson’s ratio: soil	0.3
Poisson’s ratio: soybean	0.23
Poisson’s ratio: PLA	0.3
Shear modulus: soil (MPa)	50
Shear modulus: soybean (MPa)	63
Shear modulus: PLA (MPa)	10.4
Coefficient of restitution: soil-soil	0.6
Coefficient of restitution: soybean-soybean	0.3
Coefficient of restitution: soil-PLA	0.4
Coefficient of restitution: soil-soybean	0.3
Coefficient of static friction: soil-soil	0.7
Coefficient of static friction: soybean-soybean	0.39
Coefficient of static friction: soil-PLA	0.3
Coefficient of static friction: soil-soybean	0.35
Coefficient of rolling friction: soil-soil	0.225
Coefficient of rolling friction: soybean-soybean	0.17
Coefficient of rolling friction: soil-PLA	0.03
Coefficient of rolling friction: soil-soybean	0.2

#### 2.3.3. Soil bin model.

**Fig 5:**
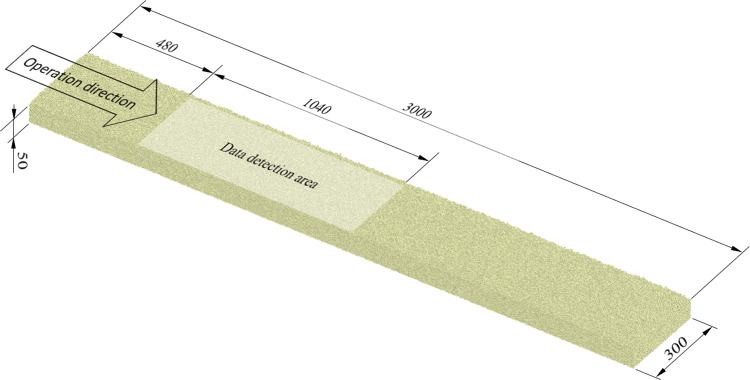
Virtual soil block and data detection area (all dimensions are in mm).

Based on the operation of the soybean plot breeding planter in the field and the need for a simulation test to determine the size of the soil block model, with a length of 3000 mm and a width of 300 mm, the soil height should be at least 40 mm. Uncovered geometries with dimensions of 3000 mm in length, 300 mm in width, and 150 mm in height were created as earth slots in EDEM software. A rectangular particle factory, measuring 3000 mm x 300 mm, was created on top of this geometry. The particle factory generated a total of 640,000 soil particles, which were leveled to form a 3000 mm x 300 mm x 50 mm soil block. The 480 ~ 1520 mm working section where the furrow opener and particle movement have been stabilized is selected as the data detection area, and the length of 1040mm can collect sufficient simulation data for analysis. The virtual soil block and data detection area are shown in Fig 5.

#### 2.3.4. Operational parameter settings.

**Fig 6:**
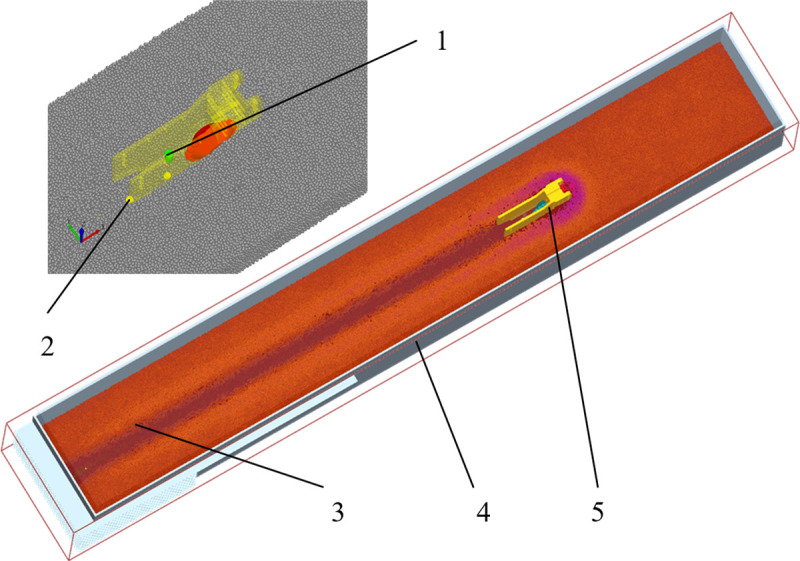
Simulation of the operating process of a furrow opener. 1. Particle factory 2. Soybean 3. Soil block 4. Soil bin 5. Sharp-angle spherical roller furrow opener.

Set the furrow opener’s forward speed to 1000 mm/s. Add a particle factory to the seed guide tube of the furrow opener and set the seed spacing to 50 mm (the particle factory generates a seed particle every 0.5s in the working phase) to mimic the seed discharging process of the seed dispenser. The three types of furrow openers are divided into three groups, respectively: the roller group, the pointed-bottom group, and the flat-bottom group; each group has the same conditions, except for different models. The Simulation of the operating process of the furrow opener is shown in Fig 6.

### 2.4. Conditions and methods of comparative testing of the soil bin

**Fig 7:**
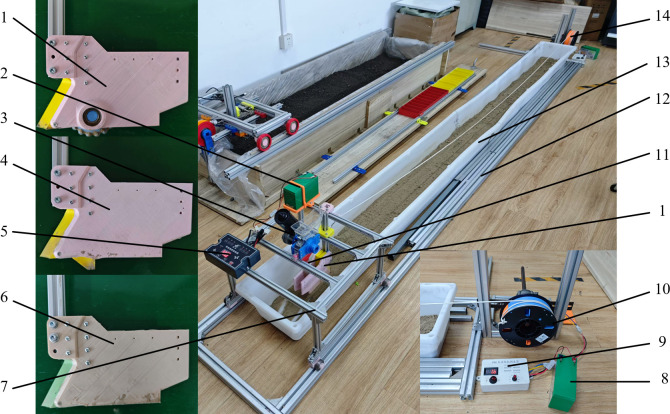
Schematic Diagram of Furrow Opener Soil Bin Comparative Test. 1. Sharp-angle spherical roller furrow opener 2. Battery for seed-metering device 3. Seed metering device 4. Sharp-angle pointed-bottom furrow opener 5. Controller for seed-metering device 6. Sharp-angle flat-bottom furrow opener 7. Track-mounted trolley (furrow opener can be installed) 8. Battery for traction device 9. Controller for traction device 10. Electric winch 11. Seed guide tube 12. Track for track-mounted trolley 13. Soil bin 14. Homemade traction device.

The three furrow openers were machined and assembled using 3D printing technology. A comparison test was conducted using the homemade test soil bin, which had dimensions of 3800 mm in length, 300 mm in width, and 1500 mm in height. The soil in the soil bin is river silt-sand from Huai’an City, Jiangsu Province, with a measured moisture content of 21.3%. The three openers and the homemade test soil bin are shown in Fig 7.

To facilitate a comparison of the effects of different seed furrows on seeding uniformity and precision, the depth of the furrow opener into the soil was uniformly adjusted to 50 mm. To ensure the stable performance of the seed planter during the experiment, the HeNong 60 soybean seeds used in the experiment were cleaned and graded in size to obtain seeds with highly consistent physical properties. This ensures that the soil does not flow back to cover the seed after the furrow is opened. The speed of the track-mounted trolley was set to 1.44 km/h by the controller, and the theoretical seeding spacing of the soybeans was set to 100 mm by the controller for the seed-metering device. As in the simulation, the three furrow openers were divided into three groups: the roller group, the pointed-bottom group, and the flat-bottom group, and each group was tested three times. To ensure that the soil conditions in the experimental soil bin were as consistent as possible for each test, soil conditioning was performed in the soil bin before each test.

After each test, a stable section of the furrow opener (approximately 1100 mm in length) was selected and measured using a steel plate ruler. To avoid the impact of missed seeding caused by the seed metering device on the experimental data, abnormal data resulting from missed seeding were excluded. This ensures the accuracy and reliability of the analysis by eliminating irregularities that could skew the results. Results of the test.

## 3. Results

### 3.1. Analysis of simulation results

#### 3.1.1. Outline of seed furrows and seed movement trajectories.

**Fig 8:**
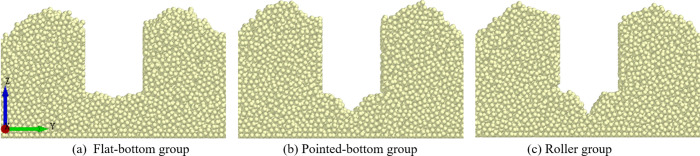
Outline of seed furrows.

Within the EDEM Analyst interface, the Clipping function enables the removal or clipping of currently uninterested model areas by defining geometric planes. By adjusting the timeline to select specific simulation timesteps, researchers can capture transient particle arrangements in the local domain. Within the EDEM Analyst interface, the Clipping function was used to clip the outline of the seed furrow opened by the three types of furrow openers, as shown in Fig 8.

**Fig 9:**
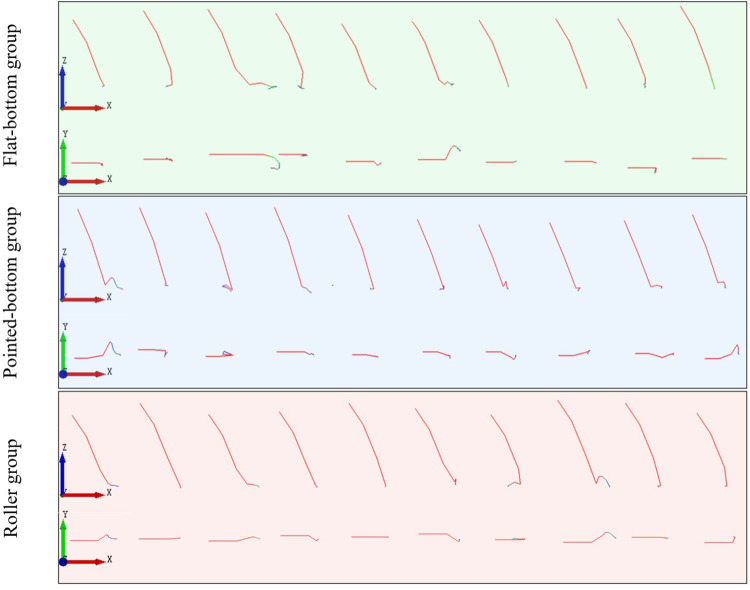
Seeds movement trajectories for each group.

To visualize the limitations of different seed furrow shapes on the displacement of seeds after touching the soil, seed movement trajectories in the XZ-plane and XY-plane were extracted from the three groups of simulations, as shown in Fig 9.

Figs 8 and 9 demonstrate that, compared to the flat-bottom group’s planar furrow base, both the pointed-bottom and roller groups feature V-shaped groove structures at the bottom of the furrow. When seeds fall into these grooves, they slide to the bottom under the influence of gravity, effectively limiting the lateral displacement of the seed. Due to the ridge-like structures formed between the depressions, the groove shape of the roller group effectively reduces the longitudinal displacement of the seed after touching the soil. In contrast, both the pointed-bottom and flat-bottom groups lack such structures, resulting in varying degrees of longitudinal displacement after seed-soil contact.

#### 3.1.2. Processing of simulation results.

In EDEM, the coordinates of 21 soybean seeds from the stable working sections of three different furrow openers were extracted. To assess the effect of openers on seed distribution uniformity and stability, this study employed the mean (absolute) lateral offset, seed spacing standard deviation, and lateral offset standard deviation (based on absolute deviation) as experimental indicators. The effect of different furrow shapes on seeding uniformity was also analyzed with graphs.

The seed spacing standard deviation directly quantifies the dispersion of actual spacing relative to the ideal spacing along the sowing direction, serving as a key indicator for evaluating longitudinal sowing uniformity. The mean lateral offset and lateral offset standard deviation respectively reflect the average precision and dispersion of sowing positions. Together, they comprehensively assess the lateral positioning accuracy and distribution stability of seeds.

The smaller the seed spacing standard deviation, the more effectively the coulter structure (such as the ridge-like protrusions on the V-shaped furrow bottom) suppresses longitudinal rolling and bouncing of seeds after placement, ensuring uniform plant spacing. A lower mean lateral offset indicates that the furrow profile formed by the coulter (such as the V-shaped seed guide channel) possesses excellent guiding functionality, reliably constraining seeds along the theoretical row line. A smaller lateral offset standard deviation reflects highly concentrated lateral seed distribution, indicating the seed opener strongly constrains random lateral displacement after seed deposition. This ensures good repeatability and high stability in the sowing process.

The standard deviation, rather than the coefficient of variation, was chosen to measure the variation in seed lateral and longitudinal offset. This decision was based on the fact that all data were measured in the same units, and the mean values across groups were relatively similar, allowing the standard deviation to reflect the magnitude of variation directly. The coefficient of variation is more suitable for comparing data with different units or significantly different means, whereas this study focuses on the absolute variation range of the seeds. Therefore, the standard deviation is a more reasonable choice for this study. The formulas for calculating the test indicators are as follows:


$$\sigma_{d}=\sqrt{\frac{\text{1}}{N-\text{1}}\sum_{i=\text{1}}^{N-\text{1}}{{(d}_{i}-\overset{―}{d})}^{\text{2}}}$$ (1)

$$\overset{―}{\left|\Delta x\right|}=\frac{\text{1}}{N}\sum_{j=\text{1}}^{N}\left|x_{j}-x_{\text{0}}\right|$$ (2)

$$\sigma_{\left|\Delta x\right|}=\sqrt{\frac{\text{1}}{N}\sum_{j=\text{1}}^{N}{\left(\left|x_{j}-x_{\text{0}}\right|-\overset{―}{\left|\Delta x\right|}\right)}^{\text{2}}}$$ (3)

Where: $\sigma_{d}$ is the seed spacing standard deviation, reflecting the degree of dispersion of the seed spacing distribution, mm; $\overset{―}{\left|\Delta x\right|}$ is the mean lateral offset, the mean of the absolute value of the lateral offset between the actual position of the seed and the theoretical row position, mm; and $\sigma_{\left|\Delta x\right|}$ is the lateral offset standard deviation, reflecting the degree of dispersion of the seed’s lateral offset, mm.

Where N is the total number of seeds; $\overset{―}{d}$ is the average seed spacing, mm; $d_{i}$ is the ith seed spacing, mm; $x_{j}$ is the actual transverse coordinate of the jth seed, mm; and $x_{\text{0}}$ is the theoretical row line transverse coordinate, mm.

**Fig 10:**
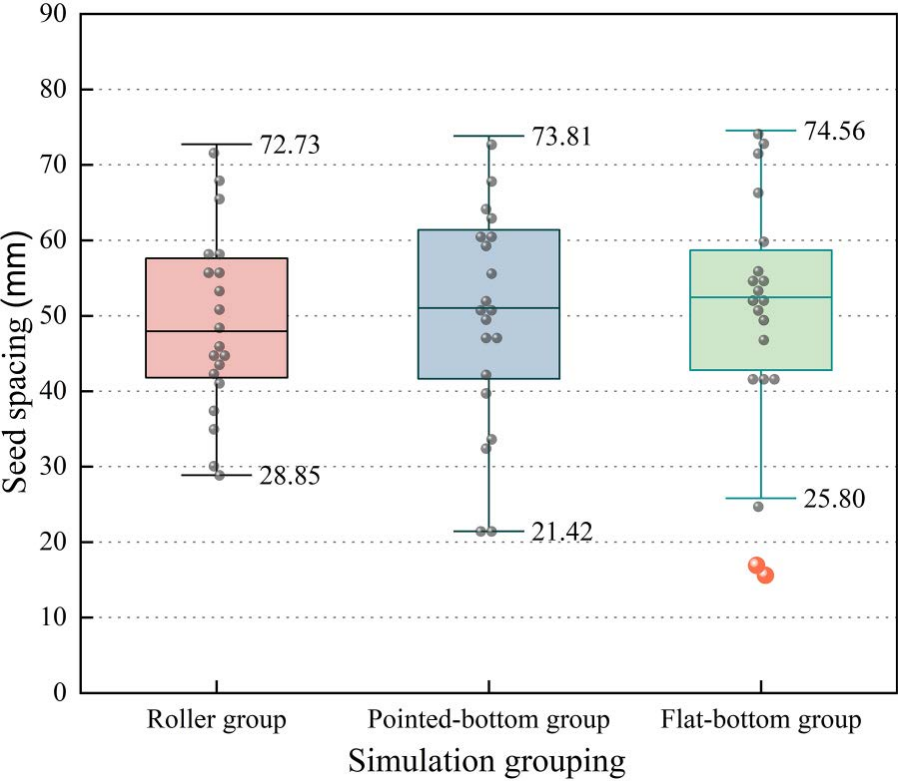
Comparative box plot of seed spacing for each group.

To quantify the effect of different furrow openers on seed spacing and lateral offset, the extracted data were statistically analyzed. Box plots of seed spacing and lateral offset for various types of furrow openers, as shown in Fig 10, were generated using OriginPro 2025 software.

From Fig 10, it can be observed that the horizontal lines representing the medians within the three boxes are all close to the standard seed spacing of 50 mm, indicating that the distributions are essentially unbiased. The box and whisker lines are the longest in the pointed-bottom group, and the box and whisker lines are the shortest in the roller group, suggesting that the data in the pointed-bottom group are more dispersed. In contrast, those in the roller group are more concentrated.

The flat-bottomed group has a wide range of variation and two lower outliers. By observing the simulation process, it was found that the flat-bottom furrow opener creates a seed furrow with a flat bottom. When the seed is released from the seed guide tube, it has forward inertia, and the relatively flat bottom of the seed furrow makes it difficult to restrict seed rolling. As a result, the spacing between two neighboring seeds can become either too large or too small.

**Fig 11:**
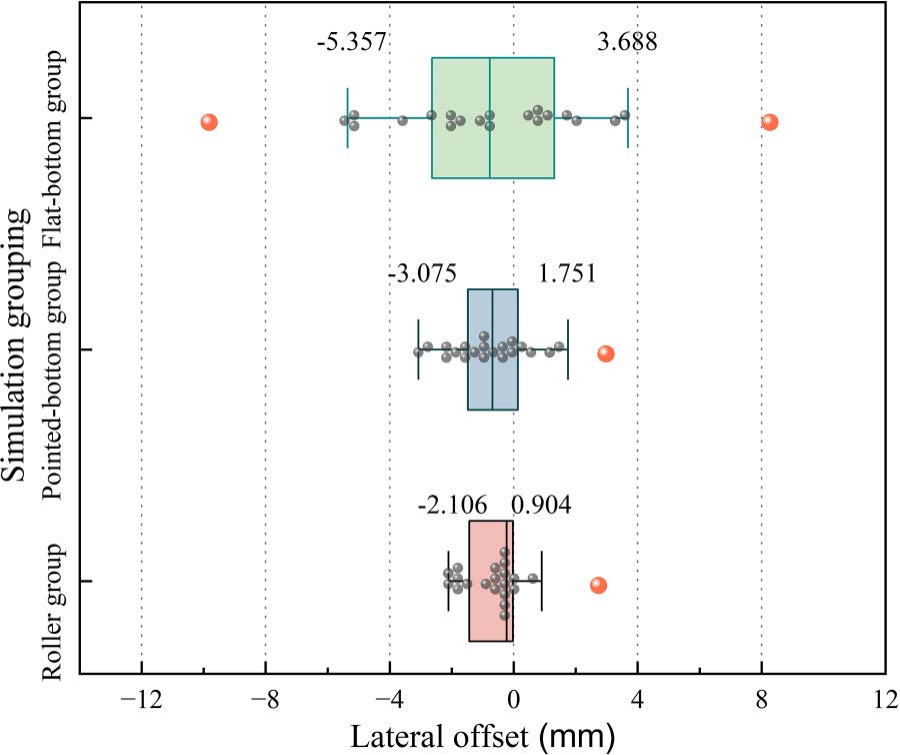
Comparative box plots of lateral offset for each group.

Fig 11 shows that the flat-bottom group exhibits two larger outliers, with the longest box and whiskers showing the highest degree of data dispersion. The pointed-bottom group has a shorter box and whiskers, with data more evenly distributed between the whiskers. In contrast, the roller group has the shortest box and whisker plot among the three groups, with the most concentrated data and a median closer to the theoretical row line position (0 mm), demonstrating the best control over seed lateral offset.

The reasons for the presence of outliers in the three groups were identified by viewing the simulation process. The outliers in the flat-bottom group are primarily due to the flat-bottomed furrow created by the furrow opener, which fails to restrict the bouncing and rolling of seeds after they make contact with the soil at an initial velocity. This structural limitation results in significant seed movement and variability in lateral offset. In contrast, the outliers in the roller group and pointed-bottom group are mainly caused by the gradual soil slippage that occurs after the furrow openers create the furrow. As a result, some of the seeds showed relatively significant horizontal offset. These two groups provided better control over the horizontal offset of the seeds compared to the flat-bottomed group.

**Fig 12:**
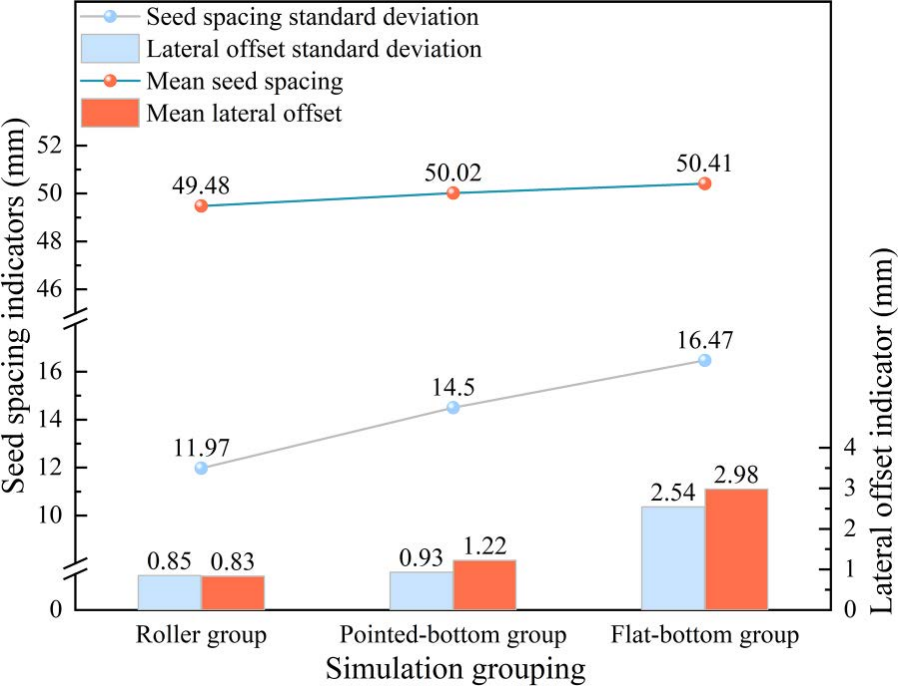
Performance comparison of different furrow openers in seed spacing and lateral offset.

**Fig 13:**
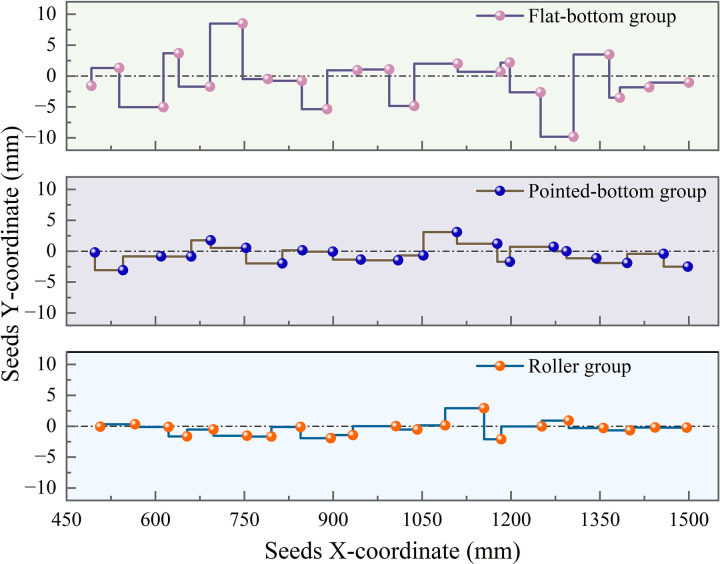
Map of the XY plane position distribution of soybean seeds.

To quantify the degree of influence of different furrow openers on seed spacing and lateral offset, relevant data were calculated and plotted to illustrate the performance of varying furrow openers in terms of seed spacing and lateral offset. This is shown in Fig 12. The extracted data were plotted as a map of the XY plane position distribution of soybean seeds to visualize the effect of the three different types of furrow openers on seed uniformity, as shown in Fig 13.

As shown in Fig 12, the seed spacing standard deviation exhibited an increasing trend: roller group (11.97 mm) <pointed-bottom group (14.5 mm) <flat-bottom group (16.47 mm). This indicates that the Flat-bottom group has the worst seed spacing uniformity while the roller group has the best seed spacing uniformity among the three groups. In terms of lateral offset, the flat-bottom group had the highest mean lateral offset (2.98 mm) and lateral offset standard deviation (2.54 mm). This indicates that the lateral position of their seeds was shifted by a large magnitude and lacked stability. The Roller group had the lowest mean lateral offset (0.85 mm) and the lowest lateral offset standard deviation (0.83 mm), indicating superior precision and stability in controlling the seeding position.

Fig 13 effectively reflects the above analysis. That is, different furrow shapes had significant effects on seeding uniformity and positional accuracy. The sharp-angle spherical roller furrow opener demonstrated the best performance in terms of spacing stability and limiting lateral displacement among the three furrow openers. However, the reliability of the simulation results needs to be further verified by soil trench tests.

### 3.2. Analysis of experimental results of soil bin

**Fig 14:**
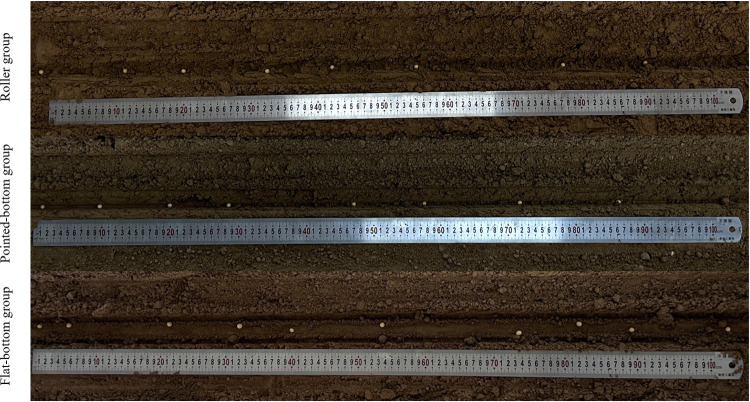
Images of soil bin tests for each group.

The operational results of different furrow openers are presented in Fig 14.

Identical experimental indicators were adopted for both physical and simulation tests: mean (absolute) lateral offset, seed spacing standard deviation, and lateral offset standard deviation (based on absolute deviation). The effect of different furrow shapes on seeding uniformity was analyzed using these three indicators, along with the necessary graphs. The relevant data were counted, and the experimental indexes were calculated. Performance indicators for seed spacing and lateral offset for each group are shown in Table 2.

**Table 2:** Performance indicators for seed spacing and lateral offset for each group.

Performance indicators	Roller group	Pointed-bottom group	Flat-bottom group
Mean lateral offset (mm)	0.58	1.36	5.15
Lateral offset standard deviation (mm)	1.09	1.19	3.09
Mean seed spacing (mm)	103.17	105.63	108.3
Seed spacing standard deviation (mm)	14.78	20.05	29.34

Although the actual experiments endeavored to replicate the conditions of the simulation tests, certain discrepancies persisted in the experimental results. The reasons for these differences can be summarized as follows:

(1) The settings of contact parameters in simulation tests, the simplification of seed and soil particle models, the simplification of material properties, and the assumption that the furrow opener and soil bin behave as rigid bodies. These factors inevitably introduce discrepancies from the real world.
(2) In simulation trials, soybean seeds and soil particles are generated under fixed conditions via the ‘particle factory’. In the real world, however, the initial state of seeds dispensed by seeders will exhibit some variation, and achieving complete uniformity in soil conditions is exceedingly difficult.
(3) Simulation trials can directly extract target seed coordinates via software with exceptional precision. Conversely, field trials involve manual measurements in soil after trenching, where accuracy is constrained by the minimum scale division of measuring instruments and may be subject to the subjective judgment of the measurer.

However, the two tests reached consistent conclusions on the differences in the key performance indicators of the three openers.

**Fig 15:**
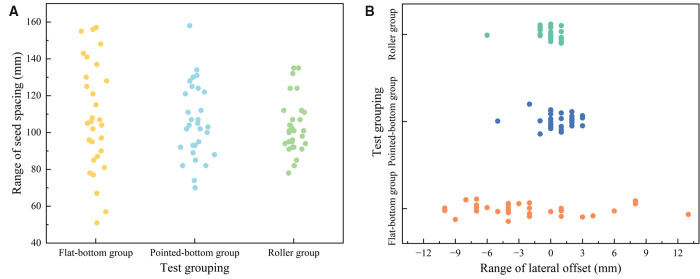
Scatterplot of the range of seed spacing and lateral offset for each group. a. Scatterplot of the range of seed spacing for each group. b. Scatterplot of the range of lateral offset for each group.

As shown in Table 2 and Fig 15, the roller group demonstrated better performance in terms of lateral offset and seed spacing control. Compared with the pointed-bottom and flat-bottom groups, the roller group had the lowest standard deviation in lateral offset and seed spacing and a more stable distribution of seeds within the preset range.

## 4. Conclusion

To improve the uniformity of seeding in soybean plot breeding planter tests, thereby avoiding inaccurate test results caused by uneven seeding. A sharp-angle spherical roller furrow opener was designed to improve the stability of sowing spacing and, at the same time, reduce the lateral offset of the seeds, and the main research conclusions are as follows:

(1) To verify the relevant performance of the designed sharp-angle spherical roller furrow opener, the mean (absolute) lateral offset, lateral offset standard deviation, and seed spacing standard deviation were selected as test indicators. A comparative discrete element simulation test was conducted with the sharp-angle flat-bottom furrow opener and the sharp-angle pointed-bottom furrow opener. The simulation results showed that compared to the sharp-angle flat-bottom furrow opener and the sharp-angle pointed-bottom furrow opener, the sharp-angle spherical roller furrow opener reduced the seed spacing standard deviation by 17.45% and 27.32%, respectively, the lateral offset standard deviation by 8.60% and 66.54%, and the mean lateral offset by 31.97% and 72.15%, respectively, stabilizing lateral offset within the range of −2.106 to 0.904 mm.
(2) Utilizing 3D printing technology, three types of furrow openers were manufactured and assembled onto a Track-mounted trolley to conduct tests. The test aimed to validate the improvement in sowing uniformity achieved by the sharp-angle spherical roller furrow opener compared to the other two furrow openers. The test results demonstrated that the pointed-angle roller furrow opener reduced the seed spacing standard deviation by 10.36% and 30.86%, respectively, when compared to the sharp-angle flat-bottom furrow opener and the sharp-angle pointed-bottom furrow opener. Additionally, it reduced the lateral offset standard deviation by 8.40% and 64.72%, respectively, and decreased the mean lateral offset by 88.47% and 57.35% compared to the other two furrow openers.
